# Effects of Ellagic Acid on Angiogenic Factors in Prostate Cancer Cells

**DOI:** 10.3390/cancers5020726

**Published:** 2013-06-19

**Authors:** Luca Vanella, Claudia Di Giacomo, Rosaria Acquaviva, Ignazio Barbagallo, Giovanni Li Volti, Venera Cardile, Nader G. Abraham, Valeria Sorrenti

**Affiliations:** 1 Department of Drug Science, Section of Biochemistry, University of Catania, I-95125 Catania, Italy; E-Mails: lvanella@unict.it (L.V.); cdigiaco@unict.it (C.D.G.); racquavi@unict.it (R.A.); barbagallo@unict.it (I.B.); livolti@unict.it (G.L.V.); 2 Department of Bio-Medical Sciences, Section of Physiology, University of Catania, I-95125, Catania, Italy; E-Mail: cardile@unict.it; 3 Joan C. Edwards School of Medicine, Marshall University, Huntington, WV 25701, USA; E-Mail: abrahamn@marshall.edu

**Keywords:** prostate cancer, angiogenesis, heme-oxygenase, cytochrome P450

## Abstract

*Background*: Several natural antioxidants, including ellagic acid (EA), have been reported to have chemotherapeutic activity *in vivo* and *in vitro* settings. Cytochrome P450 (CYP) activity and synthesis of both epoxyeicosatrienoic acids (EETs) and 20-hydroxy-5,8,11,14-eicosatetraenoic acid (20-HETE), together with vascular endothelial growth factor (VEGF) and heme oxygenase system (HO) have emerged as important modulators of tumor growth and metastasis. *Methods*: The anti-angiogenic effects of EA were investigated in the human prostatic cancer cell line LnCap. HO-1, HO-2, CYP2J2 and soluble epoxyde hydrolase (sEH) expressions were evaluated by western blotting. Levels of VEGF and osteoprotegerin (OPG) were determined in the culture supernatant using an ELISA assay, while CYP mRNAs were determined by qRT-PCR. *Results*: EA treatment induced a significant decrease (*p* < 0.05) in HO-1, HO-2 and CYP2J2 expression, and in VEGF and OPG levels. Similarly CYP2J2, CYP4F2 and CYPA22 mRNAs were significantly (*p* < 0.05) down-regulated by EA treatment. The decrease in CYP2J2 mRNA was associated with an increase in sEH expression. *Conclusions*: Results reported in the present study highlighted the ability of EA to modulate a new pathway, in addition to anti-proliferative and pro-differentiation properties, via a mechanism that involves a decrease in eicosanoid synthesis and a down-regulation of the HO system in prostate cancer.

## 1. Introduction

Prostate cancer (PC) is one of the most common male cancer in the Western world and it is considered the most rapidly increasing cancer diagnosed in Japanese men [1,2]. Prostate cancers typically start as androgen-sensitive lesions but frequently develop into androgen-insensitive lesions with the progression to advanced stages. The development and progression of PC involve various and complex factors and novel preventive approaches are needed to prevent this disease. Chemoprevention by naturally occurring dietary substances might represent a preventive approach [3]. Epidemiological evidences strongly suggest that dietary habits such as the use of fruits and vegetables may have significant impact on the development of prostate cancer. Pigmented and tropical fruits and vegetables have been shown to contain many polyphenols, which contribute to their strong antioxidant and anti-inflammatory properties [4]. Among natural polyphenols, 2,3,7,8-tetrahydroxy[1]-benzopyrano[5,4,3-cde][1]benzo-pyran-5,10-dione (ellagic acid, EA) is found, as both free and bound forms, in numerous fruits and vegetables, especially in raspberries, strawberries, nuts and in the pomegranate; it has been previously shown to possess anti-inflammatory, anti-oxidant and anti-carcinogenic properties such as inhibition of tumor formation and growth both *in vitro* and *in vivo* models [5,6,7,8,9,10,11,12,13,14,15,16,17,18].

The potential chemopreventive effects of EA have been attributed to various mechanisms including growth-inhibition and apoptosis promoting activity in several cancer cell lines [8,12,19,20]. Our previous study have demonstrated a dose-dependent cytotoxic effect of EA, which also caused a reduction in proliferation rate and marked increase in DNA damage in prostatic cancer cell lines [21]. Moreover, treatment with EA induced a reversion of prostatic cancer cell lines from a proliferating to a differentiated state [21]. 

Despite significant improvements in local and systemic therapies, most deaths from prostate cancer are due to metastasis, which are resistant to conventional therapies [22,23,24]. Tumor growth and metastasis formation depend upon development of a neo-vasculature around the tumor [25,26,27]. This process, named angiogenesis, can be considered a major factor affecting the metastatic spread of cancer cells. Angiogenesis is a multistep process, regulated by a balance between stimulatory and inhibitory factors released by the tumor and its microenvironment [22,28,29]. Therefore, angiogenesis process could be an important target to suppress tumor growth and metastasis. Angiogenesis is required at almost every step of tumor progression and metastasis, and tumor vasculature has been identified as strong prognostic marker for tumor grading [30]. Thus, inhibition of angiogenesis may represent a promising therapeutic strategy for cancer. Many phytochemicals could have a potential as anti-angiogenic agents able to control cancer development and metastasis [30].

EA exhibited pharmacological effects in various angiogenesis-dependent diseases such as diabetic retinopathy [31,32] and recently Wang *et al.* [33] reported that it exerts potent anti-angiogenesis activities via specifically targeting VEGFR-2 and its signaling pathway in breast cancer.

However, other mechanisms responsible for such effects have to be explored. Heme oxygenase (HO) catalyzes the initial, rate-limiting step of heme degradation to generate free iron, carbon monoxide (CO) and biliverdin. HO-derived CO plays a key role in the regulation of vascular tone while biliverdin is subsequently converted by biliverdin reductase to bilirubin, a potent endogenous antioxidant. 

The HO system (HO-1 and HO-2) has emerged as a fundamental endogenous cytoprotective and anti-inflammatory system. HO-1 is an inducible enzyme, whereas HO-2 displays, in general, a constitutive expression that may be altered in some human pathological conditions [34]. The role of the constitutive isoform HO-2 is largely unexplored. HO-2 is constitutively expressed in all tissues and is the main basal source for the putative mediators of the HO cytoprotective function, *i.e.*, CO and biliverdin/bilirubin. The role of HO system in cancer stems from the demonstration that HO is a potent regulator of cell growth and angiogenesis [35]. To this regard, Sunamura *et al*. also showed that HO-1 is responsible for accelerating tumor angiogenesis in human pancreatic cancer [36]. Furthermore, Botros *et al*. have demonstrated that HO-1 induction participates in the regulation of arachidonic acid metabolism (AA), which is implicated in several physiological and pathological processes, including cancer progression [37]. The AA metabolism by CYP enzymes in various cell types can be divided into two major categories, *i.e.*, hydroxylases producing 20-HETE, and epoxygenases catalyzing the formation of EETs. EETs, as well as 20-HETEs, play critical roles in cell proliferation, vasodilation and inflammation. Numerous proteins of the CYP2C family are overexpressed in malignant prostate tissue samples. 20-HETE synthases have been reported to be highly expressed in cell lines and tumor tissues (including prostate cancer, hepatoma, renal carcinoma, lung carcinoma) [38]. In addition, 20-HETE levels were increased in urine samples from patients with prostate cancer [39]. Since phytochemicals are potential novel lead compounds for developing anti-angiogenic drugs [40,41], the goal of the present study was to examine the possible molecular mechanisms underlying the anti-angiogenic properties of EA in prostate cancer with particular regards to growth factors and enzymatic machinary involved in prostate cancer progression. 

## 2. Results and Discussion

**Figure 1:**
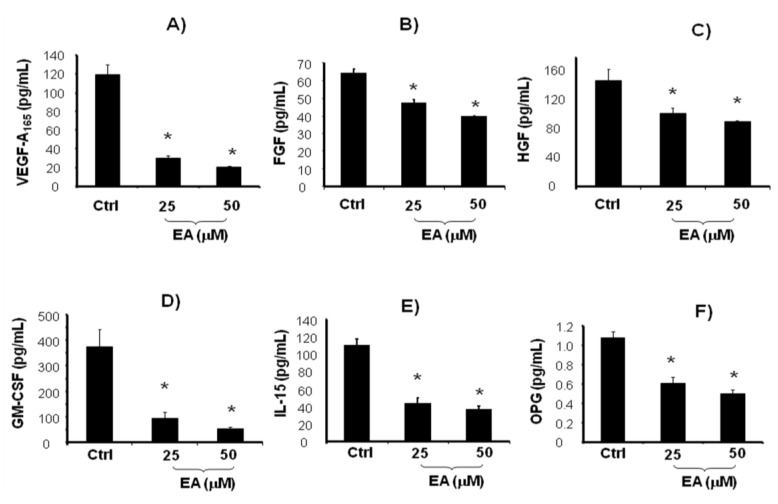
VEGF-A_165_, FGF, HGF, GM-CSF, IL-15, and OPG (**A**–**F**) levels in LnCaP cells untreated and treated for 48 h with EA at different concentrations (25 and 50 μM). Values represent the means ± SD of 4 experiments performed in triplicate. * Significance *versus* untreated control cells: *p* < 0.05.

In recent years, naturally occurring antioxidant compounds present in the human diet have gained considerable attention as cancer chemo-preventive and chemotherapeutic agents [42,43,44]. Our previous study [21] demonstrated that the natural polyphenol EA may be considered a promising cancer therapeutic agent, with both anti-proliferative and pro-differentiation properties. Angiogenesis involves a sequence of coordinated events initiated by the expression of angiogenic factors with subsequent binding to their receptors on endothelial cells. VEGF, the most important angiogenic signal protein, stimulates tumor neo-angiogenesis by increasing mitogenic and survival properties of vascular endothelial cells [45,46]. The neovascularization is necessary for the growth of solid tumors, invasion and metastasis. VEGF represents the prominent angiogenic factor that promotes the proliferation, survival, migration and tube formation of endothelial cells. We then examined, in untreated and EA treated LnCap cells, the levels of VEGF and others growth factors involved in prostate cancer progression such as Fibroblast growth factor (FGF), Granulocyte colony-stimulating factor (G-CSF), Hepatocyte growth factor (HGF) [47,48,49]. Moreover, since chronic inflammation has been suggested to favour prostate cancer development [50], the levels of Interleukin 15 (IL-15) were measured. EA treatment (25–50 μM) induced a significant decrease of VEGF, FGF, G-CSF, HGF and IL-15 levels compared to untreated cells (Figure 1A–E).

**Figure 2:**
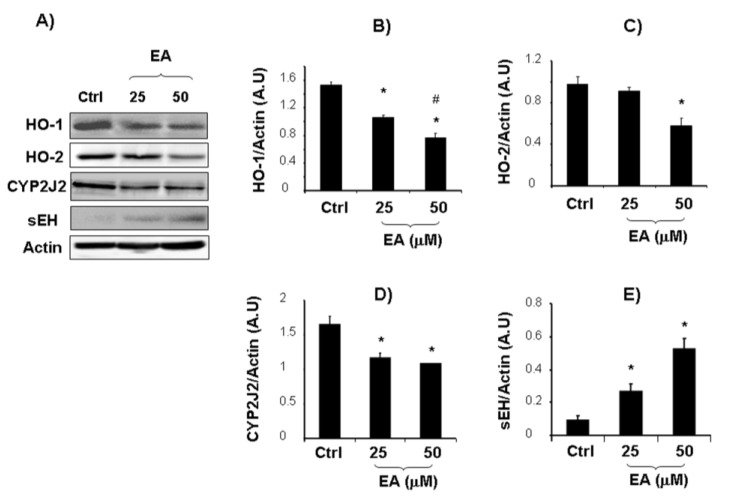
Effect of EA (25 and 50 μM) on HO-1, HO-2, CYP2J2 and sEH expressions in cultured LnCap cells (**B–E**). Results, expressed as arbitrary units (AU), represent the mean ± SD of 4 experiments performed in triplicate. Significance of 25–50 μM EA *versus* control; * *p* < 0.05. Significance of 50 μM EA *versus* 25 μM EA; # *p* < 0.05. Representative Western blotting of HO-1, HO-2, CYP2J2 and sEH protein expression in cultured LnCap cells (**A**).

Invasive prostate cancer is often associated with bone metastases and with an increase in OPG levels [51,52]. Serum OPG has been shown to be a reliable marker in detecting bone metastatic spread and a predictor of mortality from prostate cancer [53]. Thus, we further examined whether EA affects OPG levels released by LnCap cells. ELISA analysis showed decreased levels of OPG after EA treatment (25–50 μM) compared with untreated cells (Figure 1F). The decreased levels of OPG, IL-15, VEGF and the above cited growth factors, support the hypothesis that EA treatment may contribute to reduce tumor-related angiogenesis and metastasis. Several reports link VEGF with cancer, probably via interactions with heme oxygenase (HO), suggesting an involvement of HO in multiple pathways including regulation of cell proliferation and angiogenesis [35,54,55]. HO-1 expression has been demonstrated to be associated with prostate cancer progression [56]. In addition, it has been shown that selective inhibition of HO-2 by siRNA increases reactive oxygen species and activates caspases inducing apoptotic cell death [57], but up to date, the role of HO-2 in cancer is almost unexplored. Results reported in the present study evidenced, for the first time, the ability of EA to decrease the expressions of HO-1 and HO-2, both representative of one of the most important cytoprotective system in the cell. Although HO-1 is known to be modulated by different molecules in malignant tissue, the decreased expression of HO-2 was unexpected, as this isoform is constitutively active. According to He *et al.* [57], HO-2 reduction may contribute to the activation of apoptotic pathway and inhibition of cell growth. A down-regulation of the HO system could contribute to tumor regression by decreasing HO-1/2-derived CO, which is involved in tumor angiogenesis (Figure 2B,C). 

The interactions between HO system, AA and its metabolites have stimulated great interest in vascular biology and were originally studied in relation to inflammatory and cardiovascular diseases [58]. On the other hand, the role of cytochrome P450-derived eicosanoids, such HETEs and EETs remains poorly characterized in cancer biology. 

Since HO-1 has been shown to increase EETs levels and *vice versa* [59] we examined whether EA treatment modulate AA in prostatic cancer cells. The AA pathway has been shown to play a major role in the development and progression of a number of cancers, including prostate cancer [60]. Free AA is metabolized via cyclooxygenases, lipoxygenases, or CYP450 oxygenases to bioactive molecules such as prostaglandins, thromboxanes, leukotrienes, and CYP450-derived metabolites [61]. Epoxidation of arachidonic acid to EETs is catalyzed by a large number of CYP450 isoforms [62]. The effects of EETs and VEGF regulation are closely inter-linked. EETs can enhance the effects of VEGF-induced angiogenesis [63,64,65]. Furthermore, it has been demonstrated that elevated activity of CYP epoxygenases, including CYP2J2, could promote cancer growth, metastasis [66,67] and tumor angiogenesis [68,69]. In particular, it has been reported that LnCap cells overexpress CYP2J2 [70]. Upon formation, EETs are subjected to rapid hydrolysis by the sEH to their respective, less active, dihydroxyecosatrienoic acids (DHETEs). sEH is a bi-functional enzyme with a C-terminal epoxide hydrolase activity and an N-terminal phosphatase activity. 

**Figure 3:**
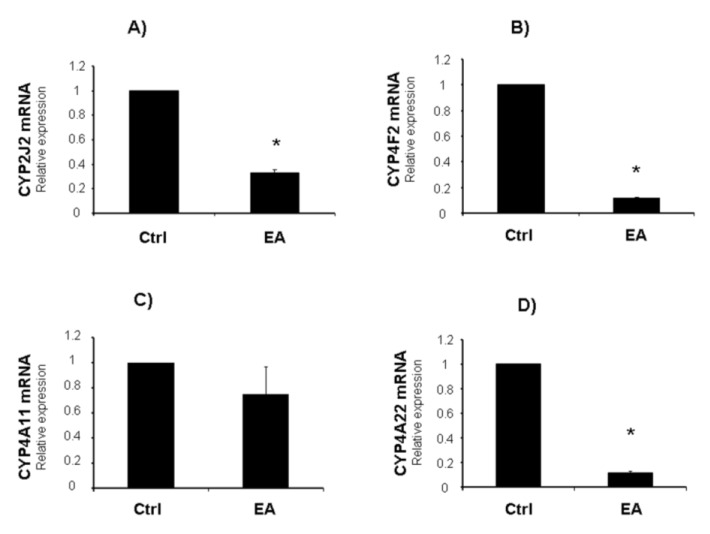
Effect of EA (50 μM) on CYP2J2, CYP4F2, CYP4A11 and CYP4A22 mRNA levels in cultured LnCap cells (**A–D**). Results represent the mean ± SD of 4 experiments performed in triplicate. Significance of 50 μM EA *versus* control; * *p* < 0.05.

Figure 2D,E report densitometric analysis of CYP2J2 and sEH expression in untreated and EA treated LnCap cells. EA treatment induced a significant increase of sEH and a decrease of CYP2J2 protein expression in EA treated cells. Consistently, mRNA levels of CYP2J2 were decreased after EA treatment (Figure 3A).

As a result of these effects, EA might reduce angiogenesis through lowering EET synthesis levels and increasing their degradation. As reported in our previous study, prostate cancer cell lines [21], as well as prostate cancer tissues [71], show increased expression of DNA methyltransferase 1 (DNMT-1) compared to normal prostatic epithelial cells. We reported that the anti-proliferative effects of EA may be related to reduction of DNMT-1 expression. It has been reported that DNA methylation could be an important epigenetic mechanism leading to a decrease of sEH levels in hepatocellular carcinoma cells [72]. The results reported here extend our previous findings, confirming that EA treatment is capable of inhibiting cancer cell proliferation via the AA signaling pathway. Interestingly, EA treatment affected not only the epoxydation of AA, but also its hydroxylation that it is responsible for the formation of 20-HETEs. Emerging evidence suggest that 20-HETEs promote pathological angiogenesis in various disease states such as cancer, atherosclerosis and diabetes. Endothelial cell (EC) proliferation is one of the early steps in angiogenesis and cancer growth. 20-HETE was shown to induce the proliferation of human EC *in vitro* via stimulating ROS formation and the production of VEGF [73]. 20-HETE plays a critical role in angiogenic responses via regulating the proliferation, migration, tube formation, and survival of both EC and VSMC [74]. A previous study demonstrated that a 20-HETE antagonist (WIT002) inhibited the proliferation of renal adenocarcinoma [75]. These findings are consistent with our hypothesis and results, showing that EA (50 μM) down regulated CYP4F2 and CYP4A22 mRNA, which represent the main CYP isoforms producing 20-HETEs. CYP4A11 mRNA levels were not significantly altered after EA treatment (Figure 3B–D). EA (25 μM) did not induce any significant modification in mRNA levels (data not shown). Our study provides experimental evidence that inhibiting 20-HETEs formation may offer a strategy to reduce pathological angiogenesis. CYP4A/F-20-HETE system merits consideration as a new therapeutic target to modulate angiogenesis in cancer progression.

## 3. Experimental Section

### 3.1. Cell Culture and Treatments

LnCap frozen cells were purchased from American Type Culture Collection (Rockville, MD, USA). After thawing, LnCap cells were resuspended in RPMI 1,640 medium (Sigma-Aldrich, St. Louis, MO, USA), supplemented with 10% heat inactivated fetal bovine serum (FBS, Invitrogen, Carlsbad, CA, USA) and 1% antibiotic/antimycotic solution (Invitrogen). Cells were plated at a density of 1–5 × 10^6^ cells per T75 flask. Cell cultures were maintained at 37 °C in a 5% CO_2_ incubator, and the medium was changed after 3–4 days. Sub-confluent cells were treated for 48 h with two different concentrations (25 and 50 μM) of freshly prepared EA dissolved in dimethylsulfoxide (DMSO). Control groups received DMSO alone.

### 3.2. Immunoblot Analysis

Cells were cultured in T75 flasks, washed with PBS and then trypsinized (0.05% trypsin w/v with 0.02% EDTA). The pellets were lysed in buffer (Tris-HCl 50 mM, EDTA 10 mM, Triton X-100 1% v/v, PMSF 1%, pepstatin A 0.05 mM and leupeptin 0.2 mM) and, after mixing with sample loading buffer (Tris-HCl 50 mM, SDS 10% w/v, glycerol 10% v/v, 2-mercaptoethanol 10% v/v and bromophenol blue 0.04%) at a ratio of 4:1, were boiled for 5 min. Samples (20 μg protein) were loaded into 8 or 12% SDS-polyacrylamide (SDS-PAGE) gels and subjected to electrophoresis (120 V, 90 min). The separated proteins were transferred to nitrocellulose membranes (Bio-Rad, Hercules, CA, USA; 1 h, 200 mA per gel). After transfer, the blots were incubated with Li-Cor Blocking Buffer for 1 h, followed by overnight incubation with 1:1,000 dilution of the primary antibody. Primary polyclonal antibodies directed against HO-1, HO-2 were purchased from Enzo Life Sciences (Farmingdale, NY, USA) while CYP2J2 and sEH were purchased from Santa Cruz (Dallas, TX, USA). After washing with TBS, the blots were incubated for 1 h with secondary antibody (1:1,000). Protein detection was carried out using a secondary infrared fluorescent dye conjugated antibody absorbing at 800 nm or 700 nm. The blots were visualized using an Odyssey Infrared Imaging Scanner (Li-Cor Science Tec) and quantified by densitometric analysis performed after normalization with β-actin (Santa Cruz). Results were expressed as arbitrary units (AU).

### 3.3. VEGF and OPG Measurements

Cells were seeded at a constant density to obtain identical experimental conditions in the different tests and to achieve a high accuracy of the measurements. VEGF-A_165_ and OPG levels were determined in the culture supernatant using an ELISA kit (AssayGate, Ijamsville, MD, USA). The assays were performed according to manufacturer’s guidelines. Results were expressed as pg/mL.

### 3.4. FGF, G-CSF, HGF and IL-15 Measurements

Cells were seeded at a constant density to obtain identical experimental conditions in the different tests and to achieve a high accuracy of the measurements. Levels of cytokines and growth factors (*FGF*, *G-CSF*, *HGF and IL-15*) were determined in culture supernatants at each time point by Milliplex™ MAP kit (Millipore, Billerica, MA, USA). Millipore multiscreen 96-well filter plates for multiplex cytokines and growth factors kits were used. Each sample was run in triplicate in accordance with the protocol of the manufacturer, and the final results presented here are representative of three independent experiments. Data were collected by using Luminex-100 software version 1.7 (Luminex, Austin, TX, USA), and analysis was performed with the MasterPlex QT 1.0 system (MiraiBio, Alameda, CA, USA). A five-parameter regression formula was used to calculate the sample concentrations from the standard curves. Data were analyzed by using either 5- or 4-parameter logistic or spline curve-fitting method as recommended by the manufacturer. Type of curve-fitting method was chosen for each analyte with respect to the lowest residual variance (<5%). Results were expressed as pg/mL.

3.5. mRNA Isolation and Real-Time PCR Quantification

Total RNA was isolated using Trizol^®^ (Invitrogen) according to the manufacturer’s instructions. First-strand cDNA was synthesized with Roche reverse transcription reagents. Total RNA (1 μg) was analyzed by real-time PCR. The quantitative real-time polymerase chain reaction (qRT-PCR) was performed with the SYBR® Green Master Mix (Applied Biosystems, Foster City, CA, USA) assay on an ABI Prism 7,900 sequence analyzer according to the manufacturer’s recommended protocol (Applied Biosystems). Each reaction was run in triplicate. The comparative threshold cycle (*C*_T_) method was used to calculate the amplification fold as specified by the manufacturer. A value of 10 ng of reverse-transcribed RNA samples was amplified by using the SYBR® Green Master Mix. Oligo-dT custom (SA Biosciences, Frederick, MD, USA) primers were used for human CYP2J2 (PPH01230B-200), CYP4A22 (PPH57966A-200), CYP4A11 (PPH01232E-200), CYP4F2 (PPH01218A-200). 

### 3.6. Statistical Analyses

Statistical significance between experimental groups was determined by the Fisher method of analysis of multiple comparisons (*p* < 0.05). For comparisons among treatment groups, the null hypothesis was tested by a two-factor ANOVA for multiple groups or unpaired *t* test for two groups. Data are presented as mean ± SD.

## 4. Conclusions

Further to the previously reported anti-proliferative and pro-differentiation properties of EA, the present study shows that EA may also interfere with multiple biologic processes involved in angiogenesis and metastasis of cancer cells. Modulation of proteins involved in tumor cell invasiveness and angiogenesis may represent mechanisms of cell growth inhibition and reduced neovascularization by EA. In conclusion, the results of this study demonstrate that EA treatment may represent a new approach and highly effective strategy in reducing carcinogenesis.
